# Bradycardia, Renal Failure, Atrioventricular Nodal Blockade, Shock, and Hyperkalemia (BRASH) Syndrome: A Deadly Pentad of Symptoms

**DOI:** 10.7759/cureus.106824

**Published:** 2026-04-10

**Authors:** Peter Killian, James Espinosa, Alan Lucerna

**Affiliations:** 1 Emergency Medicine, Jefferson Health, Stratford, USA

**Keywords:** acute kidney injury, atrioventricular nodal blockade, bradycardia, brash syndrome, hyperkalemia

## Abstract

Bradycardia, renal failure, atrioventricular (AV) nodal blockade, shock, and hyperkalemia (BRASH) syndrome is an underrecognized clinical entity characterized by a synergistic interplay among hyperkalemia, renal dysfunction, and AV nodal blockade. This self-perpetuating cycle can lead to profound bradycardia and hemodynamic instability if not promptly identified and managed. We report the case of a man in his 70s who presented to the emergency department (ED) with acute-onset watery diarrhea followed by a syncopal episode. On evaluation, he was bradycardic and hypotensive, with laboratory findings notable for hyperkalemia and acute kidney injury. His home medications included amiloride, carvedilol, and losartan. The electrocardiogram (ECG) demonstrated complete heart block. The patient was treated medically for hyperkalemia, with subsequent spontaneous resolution of the bradyarrhythmia. He was diagnosed with BRASH syndrome and admitted to the intensive care unit for further monitoring. In the setting of hyperkalemia and concurrent use of AV nodal blockers, there is a risk of worsening bradycardia and cardiovascular collapse if not recognized early. This case highlights the importance of early identification of BRASH syndrome in patients presenting with bradycardia, particularly those receiving AV nodal blockers. Prompt correction of hyperkalemia and supportive care may reverse the cycle and prevent progression to cardiovascular collapse.

## Introduction

The interplay among hyperkalemia, atrioventricular (AV) nodal blocking medications, and bradycardia is well established [1]. Bradycardia, renal failure, AV nodal blockade, shock, and hyperkalemia (BRASH) syndrome represents a clinical entity in which these factors interact in a self-amplifying cycle. Hyperkalemia reduces the resting membrane potential of cardiac myocytes, impairing excitability and conduction, while AV nodal blocking agents (such as beta-blockers and non-dihydropyridine calcium channel blockers) further slow conduction through the AV node. Together, these processes can produce more profound bradycardia than either factor alone, as demonstrated in animal studies and case reports [1].

Bradycardia, in turn, reduces cardiac output and renal perfusion, leading to acute kidney injury. This worsens hyperkalemia by impairing potassium excretion, further exacerbating conduction abnormalities. This positive feedback loop, linking bradycardia, renal dysfunction, and hyperkalemia, is a defining feature of BRASH syndrome and explains how initially modest abnormalities can rapidly progress to hemodynamic instability.

This cluster of findings has been described as BRASH syndrome, first clearly characterized in 2016 [1-3]. The syndrome is marked by synergistic toxicity, in which relatively mild elevations in potassium can produce significant conduction disturbances in patients receiving AV nodal blocking agents. This distinguishes it from isolated hyperkalemia or medication overdose.

As noted by Farkas et al., many experienced clinicians have likely managed BRASH syndrome without explicitly recognizing the entity [1]. However, increasing awareness is important, as the syndrome has distinct epidemiologic patterns, risk factors (particularly in older patients with underlying renal dysfunction and polypharmacy), and management considerations that differ from those of its individual components [1].

This case highlights the dynamic clinical presentation of BRASH syndrome, including the rapid progression of conduction abnormalities, and emphasizes the importance of early recognition and targeted management in reversing the underlying pathophysiologic cycle.

This case report was presented in poster form at the Rowan-Virtua Research Day in Stratford, NJ (May 8, 2025).

## Case presentation

A man in his 70s presented to the emergency department (ED) with a one-day history of profuse, watery diarrhea. He reported a syncopal episode upon standing to use the bathroom, during which he fell forward and sustained a minor facial abrasion. He denied nausea, vomiting, fever, chills, myalgias, or recent antibiotic use. His home medications included carvedilol, losartan, and amiloride, with confirmed adherence.

On arrival, his vital signs were notable for hypotension and hypoxia: blood pressure 88/53 mmHg, heart rate 103 beats per minute, respiratory rate 10 breaths per minute, temperature 98°F (oral), and oxygen saturation 90% on room air. Shortly after arrival, his heart rate decreased to 32 beats per minute. Oxygen therapy was initiated via nasal cannula at 4 L/minute, improving oxygen saturation to 95%.

Physical examination revealed a nontoxic, well-appearing male in no acute distress. Lung auscultation was clear bilaterally without wheezes. Although initially tachycardic, he later became bradycardic with an irregularly irregular rhythm. Abdominal examination revealed a soft, non-distended abdomen with mild diffuse tenderness and no peritoneal signs. Neurologically, he was alert, oriented, and appropriately responsive.

**Figure 1:**
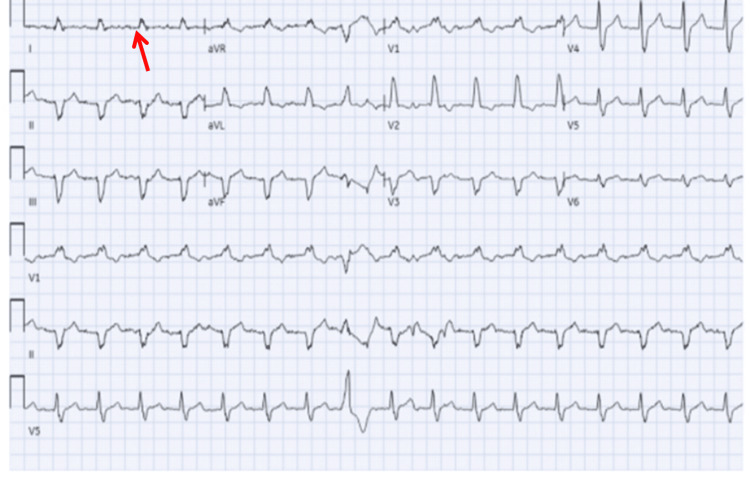
Initial ECG on arrival to the ED demonstrating sinus tachycardia with first-degree AV block (arrow) and a heart rate of 103 beats per minute. ECG: electrocardiogram, ED: emergency department, AV: atrioventricular.

An electrocardiogram (ECG) obtained on arrival demonstrated sinus tachycardia with first-degree AV block and a heart rate of 103 beats per minute (Figure 1). 

**Figure 2:**
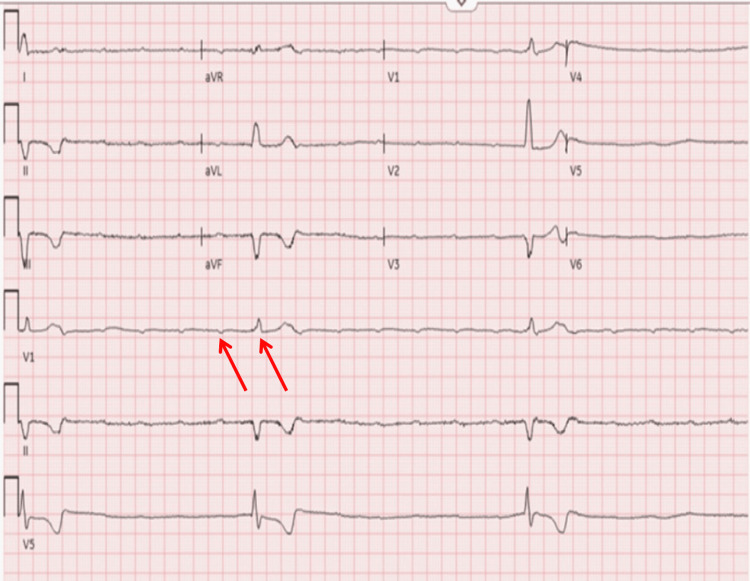
Repeat ECG demonstrating complete heart block with AV dissociation and an idioventricular escape rhythm (arrows) (ventricular rate: 36 beats per minute). ECG: electrocardiogram,  AV: atrioventricular.

A repeat ECG obtained shortly thereafter demonstrated a heart rate of 36 beats per minute, with findings consistent with third-degree AV block (Figure 2).

The patient received an intravenous fluid bolus of normal saline and 0.5 mg of atropine without clinical effect. Due to persistent bradycardia and hypotension, fentanyl 50 mcg was administered for analgesia in preparation for transcutaneous pacing. Transcutaneous pacing was then initiated.

Concurrent laboratory evaluation revealed leukocytosis (white blood cell (WBC) count of 23,500/µL), hyperkalemia (serum potassium 7.5 mEq/L), and acute kidney injury (creatinine 2.10 mg/dL) (Table 1). These findings, particularly severe hyperkalemia and acute kidney injury, represent the key laboratory abnormalities driving the BRASH physiology in this case.

**Table 1:** Laboratory investigation.

Laboratory	Result	Normal range
White blood cell count (mm^3^)	23,000	4.0-11.0
Hemoglobin (g/dL)	12.4	10.6-15.6
Platelet count (K/µL)	180	150-400
Sodium (mEq/L)	137	135-154
Potassium (mEq/L)	7.5	3.5-5
Blood urea nitrogen (mg/dL)	32	5-20
Creatinine (mg/dL)	2.1	0.6-1.2
Glucose (mg/dL)	95	70-100
Calcium (mg/dL)	8.7	8.5-10.5
Chloride (mEq/L)	101	95-105
Bicarbonate (mEq/L)	24	23-29
Magnesium (mg/dL)	1.8	1.7-2.2
Lactate (mmol/L)	2.1	0.5-2.2
Prothrombin time (sec)	11	11-13.5
Partial thromboplastin time (sec)	33	25-35
International normalized ratio	1	0.8-1.1
Total bilirubin (mg/dL)	1.2	0.1-1.2
Direct bilirubin (mg/dL)	0.3	0-0.3
Aspartate aminotransferase (IU/L)	30	8-33
Alanine aminotransferase (IU/L)	52	7-56
Alkaline phosphatase (IU/L)	183	39-117
Urine color	Clear	
Urine clarity	Clear	
Urine specific gravity	1	1.005-1.030
Urine pH	7	5-7.5
Urine glucose	Negative	
Urine protein	Negative	
Urine bilirubin	Positive	
Urine urobilinogen	Positive	
Urine ketones	Negative	
Urine blood	Negative	
Urine white cells (cells/HPF)	Negative	0-5
Urine red cells (cells/HPF)	Negative	0-5
Urine nitrite	Negative	
Urine leukocyte esterase	Negative	
Blood culture	Negative	

Immediate medical management of hyperkalemia was initiated, including intravenous calcium chloride, insulin with dextrose, and continuous albuterol nebulization.

Following medical management, the patient’s heart rate improved spontaneously, with return to sinus tachycardia and first-degree AV block. Cardiology was consulted and recommended prioritizing correction of the patient’s hyperkalemia. They advised that transcutaneous pacing could be discontinued unless bradycardia and hypotension persisted. Throughout his ED stay, the patient’s mental status remained intact. He was subsequently admitted to the intensive care unit with a working diagnosis of BRASH syndrome.

After admission to the intensive care unit, the patient remained normotensive with stable vital signs throughout his hospital course. His WBC count normalized, and blood cultures were negative. Initial and repeat lactate levels were within normal limits. There was no evidence of infection, and antibiotics were not initiated during the hospital stay. The patient experienced one episode of hypotension, which resolved with a fluid bolus. Cardiology recommended against permanent pacemaker placement, as the conduction abnormalities resolved with correction of metabolic derangements. The patient remained in the intensive care unit for two days and was then transferred to a telemetry unit. He was discharged home on hospital day 5 with close outpatient follow-up with cardiology. Carvedilol and losartan were discontinued during the hospital stay. At discharge, cardiology continued amiloride.

Taken together, this case demonstrates several key features suggestive of BRASH syndrome, including dynamic progression from tachycardia to profound bradycardia, severe hyperkalemia, acute kidney injury, and the presence of AV nodal-blocking medications in the setting of a likely precipitating factor.

## Discussion

Much of what is known about BRASH syndrome has been derived from case reports, with some recent systematic reviews [1-5]. However, these data are insufficient to establish the overall epidemiologic frequency of the syndrome. They do, however, provide insight into associated risk factors. With an aging population and increasing use of antihypertensive medications, the incidence of BRASH syndrome in older adults may rise [3]. Although the true incidence is unknown, BRASH syndrome has been described as rare [4].

Majeed et al., in a review of 70 cases, reported a mean age of 69.2 years, with an equal distribution of male and female patients [4]. A systematic review by Shah et al. demonstrated similar findings with respect to age at presentation and sex distribution [5]. The patient in this case falls within this age range.

Hypertension has been identified as the most common comorbidity (71%), followed by diabetes mellitus (48%) and chronic kidney disease (44%) [4]. Shah et al. reported similar findings and also identified atrial fibrillation and coronary artery disease as additional risk factors [5]. The patient in this case was taking carvedilol, losartan, and amiloride, with confirmed adherence.

Regarding AV nodal-blocking agents, reviews have shown that 74% of patients were taking beta-blockers, 31% calcium channel blockers, 14% amiodarone, and 3% digoxin. The use of angiotensin-converting enzyme inhibitors has also been reported [1,4-7].

The patient in this case was at increased risk, given his use of a potassium-sparing diuretic, a beta-blocker, and an angiotensin receptor blocker.

Dehydration due to diarrhea has been reported as a possible precipitating factor, along with a higher likelihood of presentation during warmer weather [8]. Although this patient did not present on a particularly warm day, he did have a one-day history of profuse watery diarrhea.

The patient presented with syncope. Syncope and fatigue were the most common presenting symptoms (49% of cases) in the review by Majeed et al. [4]. Other symptoms may reflect central hypoperfusion and include somnolence, fatigue, encephalopathy, and headache. Patients may appear relatively well despite significant abnormalities in vital signs and laboratory findings [1]. Majeed et al. reported a mean arterial pressure (MAP) of 62 mmHg and an average heart rate of 36 beats per minute [4]. In this case, the patient had a MAP of 71 mmHg and an initial heart rate of 103 beats per minute, which briefly decreased to 32 beats per minute.

The mean serum potassium reported by Majeed et al. was 5.2 mEq/L, with mild hyperkalemia (5.0-5.9 mEq/L) in 27% of cases, moderate hyperkalemia (6.0-6.4 mEq/L) in 23%, and severe hyperkalemia (>6.4 mEq/L) in 46% [4]. The patient in this case had severe hyperkalemia, with a potassium level of 7.5 mEq/L. Shah et al. reported a mean serum potassium of 6.3 mEq/L on presentation [5]. The patient’s serum creatinine was 2.1 mg/dL, which was lower than the mean values reported by Majeed et al. (3.6 mg/dL) and Shah et al. (2.3 mg/dL) [4,5].

The most common ECG finding observed by Majeed et al. was a junctional escape rhythm (50%), followed by sinus bradycardia (17%) [4]. Other findings included complete heart block (13%), AV nodal block, and pulseless electrical activity. Atrial tachycardia, atrial fibrillation, and atrial flutter were also reported [4]. Similar findings were noted by Shah et al., with junctional escape rhythm and sinus bradycardia being the most common rhythms [5]. In this patient, the ECG demonstrated complete heart block with an idioventricular escape rhythm at a rate of less than 36 beats per minute, representing an early indicator of instability.

A hallmark of BRASH syndrome is the synergistic toxicity between hyperkalemia and AV nodal-blocking agents. Even moderate elevations in serum potassium can have disproportionately severe effects on cardiac conduction in patients taking these medications. The resulting bradycardia reduces cardiac output, further impairing renal perfusion and perpetuating the cycle of renal failure and hyperkalemia. BRASH syndrome can therefore be conceptualized as a vicious cycle in which hyperkalemia leads to bradycardia, reduced cardiac output, and worsening renal function, which in turn exacerbates hyperkalemia [1,9,10].

In this case, the patient’s medication regimen included carvedilol (a beta-blocker), losartan (an angiotensin receptor blocker), and amiloride (a potassium-sparing diuretic), all of which can impair renal potassium excretion or AV nodal conduction. The development of diarrhea likely led to volume depletion and prerenal azotemia, exacerbating the hyperkalemia. This multifactorial scenario illustrates how seemingly independent factors can interact to trigger BRASH physiology.

Farkas et al. have emphasized the distinction between BRASH syndrome, AV nodal blocker toxicity, and isolated hyperkalemia. In cases of medication toxicity, there is often a history of intentional or accidental overdose. In BRASH syndrome, the response to intravenous calcium is often rapid. Isolated hyperkalemia typically does not cause significant bradycardia unless potassium levels are markedly elevated. Additionally, the presence of AV nodal-blocking medications is essential for the diagnosis of BRASH syndrome [1]. In this case, alternative diagnoses, including isolated hyperkalemia and AV nodal blocker toxicity, were considered; however, the combination of relatively modest potassium elevation relative to the severity of bradycardia, absence of overdose history, and rapid response to treatment supported a diagnosis of BRASH syndrome.

Management of BRASH syndrome requires addressing all contributing factors. A common pitfall is focusing on a single component, such as hyperkalemia alone, rather than the full clinical picture [1]. Treatment of hyperkalemia should be initiated promptly, particularly in the presence of ECG changes, such as peaked T waves, QRS prolongation, or bradycardia. Intravenous calcium stabilizes the myocardium and may improve heart rate and cardiac output. Farkas et al. recommend intravenous calcium gluconate in patients with established IV access who are not in cardiac arrest [1]. Insulin with dextrose promotes intracellular potassium shift, and nebulized albuterol may provide additional benefit. Treatment goals include correction of hyperkalemia, hemodynamic support, and management of precipitating factors such as hypovolemia or medication effects [7,11].

In this case, the initial ECG did not demonstrate classic findings of hyperkalemia, such as peaked T waves or QRS widening. This atypical presentation underscores the importance of considering BRASH syndrome in patients with unexplained bradycardia and renal dysfunction. With timely treatment, including intravenous calcium, insulin with dextrose, albuterol, and fluid resuscitation, the patient’s rhythm converted to sinus tachycardia, with stabilization of conduction.

Treatment of hyperkalemia may improve bradycardia. If bradycardia persists, Farkas et al. recommend intravenous epinephrine to improve heart rate and cardiac output. Isoproterenol may be used as a second-line agent if epinephrine is ineffective, or as a first-line agent in patients with significant bradycardia and preserved blood pressure, as normal blood pressure in this setting may reflect compensatory vasoconstriction [1].

Fluid status should be assessed based on clinical history and physical examination. If hypovolemia is present, it should be treated. A balanced crystalloid solution may be used. Normal saline should be used with caution, as large-volume administration can contribute to hyperchloremic metabolic acidosis, which may promote extracellular potassium shifts and result in a transient increase in serum potassium.

In patients with uremic metabolic acidosis, isotonic bicarbonate infusion may be considered. This approach helps correct acidemia, promotes intracellular potassium redistribution, and may improve hemodynamics. A commonly used regimen is 150 mEq of sodium bicarbonate added to 1 L of 5% dextrose in water, administered with close monitoring of volume status, serum potassium, and pH.

In patients with severe hyperkalemia who do not respond to fluid resuscitation and initial potassium-lowering therapies, high-dose diuretics may be used. Failure to respond to diuretics may indicate the need for dialysis [9].

Management of bradycardia in BRASH syndrome focuses on correction of hyperkalemia and the use of beta-agonist agents. Bradycardia may not respond to atropine, which is not considered first-line therapy once BRASH syndrome has been identified [5,10]. Transvenous pacing may be required in refractory cases.

In a review by Shah et al., 74% of patients with BRASH syndrome required admission to the intensive care unit [5]. Approximately 10% experienced cardiac arrest, and overall mortality was 7% [5]. Majeed et al. reported a mortality rate of 6% [4].

Individual case reports illustrate the variability in presentation and management. Saeed et al. described rapid stabilization in a patient requiring dialysis [12], while Certal et al. reported a case requiring 24 hours of intravenous isoproterenol in addition to calcium gluconate, fluid resuscitation, insulin, and dextrose [2]. In some cases, elevated blood glucose may initially suggest diabetic ketoacidosis [3]. The diagnostic challenge of BRASH syndrome is further illustrated in cases described by Shah et al. [13]. An association with preceding diarrhea, as seen in this case, has also been reported [14].

Roy et al. have suggested that wearable devices, such as smartwatches, may allow earlier detection of bradycardia and potentially earlier recognition of BRASH syndrome [9].

Recognition of BRASH syndrome depends on identifying a characteristic constellation of findings rather than any single abnormality. In this case, several key features are noteworthy.

First, the patient demonstrated dynamic heart rate changes, with initial tachycardia followed by rapid progression to profound bradycardia. This transition may serve as an early clinical clue to evolving BRASH physiology and should prompt consideration of a mixed or evolving process.

Second, the patient’s medication profile, including a beta-blocker, angiotensin receptor blocker, and potassium-sparing diuretic, created a physiologic substrate for impaired AV nodal conduction and reduced renal potassium excretion. This combination is commonly reported and should heighten clinical suspicion.

Third, the presence of severe hyperkalemia and acute kidney injury in conjunction with bradycardia represents the core diagnostic triad of BRASH syndrome. The electrocardiographic findings progressed from first-degree AV block to complete heart block, illustrating the range of conduction abnormalities.

Fourth, recent diarrhea likely contributed to volume depletion and prerenal azotemia, serving as a precipitating factor. Even modest volume depletion may initiate the BRASH cycle by reducing renal perfusion.

Finally, this case highlights that patients with BRASH syndrome may appear less ill than their vital signs and laboratory abnormalities suggest, underscoring the importance of integrating clinical, pharmacologic, and laboratory data.

These features provide a practical framework for identifying BRASH syndrome: bradycardia (often dynamic), renal dysfunction, hyperkalemia, use of AV nodal-blocking medications, and a precipitating factor such as dehydration or acute illness.

## Conclusions

BRASH syndrome represents a vicious cycle in which bradycardia, renal dysfunction, AV nodal blockade, shock, and hyperkalemia exacerbate one another. Early identification and targeted management, particularly correction of hyperkalemia, can result in rapid clinical improvement and, as demonstrated in this case, may obviate the need for invasive interventions.

While outcomes are favorable when promptly recognized and treated, management should be individualized, and temporizing measures such as transcutaneous pacing may be necessary, with transvenous pacing or emergent dialysis reserved for refractory cases. This case reinforces the importance of maintaining a high index of suspicion for BRASH syndrome, particularly in elderly patients on AV nodal blockers presenting with bradycardia, hypotension, and renal dysfunction. In addition, this case highlights several important clinical features, including dynamic progression from tachycardia to profound bradycardia, atypical electrocardiographic findings in the setting of severe hyperkalemia, and limited response to atropine, all of which may aid in early recognition of BRASH syndrome.
